# Clinical significance of a flattening index derived from the flow-volume loop

**DOI:** 10.3389/fphys.2026.1765817

**Published:** 2026-07-03

**Authors:** Albert Liu, Hengji Chen, Elle Commerce, Yuh-Chin T. Huang

**Affiliations:** 1 Department of Biomedical Engineering, Pratt School of Engineering, Duke University, Durham, NC, United States; 2 Department of Medicine, School of Medicine, Duke University, Durham, NC, United States

**Keywords:** asthma, bronchiolitis, CT scan, DIPNECH, emphysema, flow volume loop, pulmonary function test, small airway disease

## Abstract

**Rationale:**

An excessive decrease in the flow at the late expiratory portion of the flow volume loop (FVL) or “flattening” reflects small airway diseases (SAD). The flattening is subjectively assessed by visual inspection. In this study, we hypothesized that an objective flattening index (FI) would correlate with age and that an abnormal FI is associated with SAD.

**Methods:**

PFT reports with FEV1/FVC ≥0.7, FEV1 ≥80%pred, and FVC ≥80%pred were downloaded from electronic medical records. We digitized and converted FVLs to data points. We calculated the slopes of segment AB (between the peak expiratory flow [point A] and the point corresponding to 75% of the expiratory vital capacity [point B]) and segment BC (between point B and the point where the expiratory portion of the FVL intersects with the x-axis [point C]). Moreover, 75% is chosen because it roughly corresponds to the start of small airways. FI is the angle formed by segment AB and segment BC. We also extracted demographic data from PFT reports.

**Results:**

A total of 490 PFTs (161 men and 329 female) were included. FI was 156° ± 12° in men and 160° ± 14° in women (*p* = 0.0079). FI correlated negatively with age and BMI in both genders. The threshold value (mean-1.64 × SD) was 136°for men and 137°for women. A total of 21 PFTs (4.3%) in 15 patients had FI <threshold value. All patients had CT findings consistent with SAD and/or lung diseases involving primarily small airways.

**Conclusions:**

FI decreased with increasing age and BMI. It can be a useful parameter on PFTs to assist clinicians in identifying SAD.

## Introduction

Small airways are often considered the “quiet” zone since a disease process occurring in this region is difficult to detect clinically (Macklem and Mead, 1967; Mead, 1970). Current pulmonary function test (PFT) interpretation algorithm for airway obstruction using FEV1/FVC does not differentiate small airway disease (Stanojevic et al., 2022). Spirometry does not capture small airway function until the last part of the flow volume loop (FVL) when the expiratory flow decreases. The excessive decrease in the late expiratory flow or “flattening” in FVL may indicate the presence of small airway disease. The flattening is currently assessed by visual inspection and is subject to significant inter-rater variabilities (Hnatiuk et al., 1996; Chen et al., 2022). In a previous study, we developed an objective measure to quantify the degree of flattening (Chen et al., 2022). That study showed that the availability of a cutoff value to pulmonologist interpreters significantly improves the consistency of calling small airway disease in the PFT interpretation (Chen et al., 2022). The cutoff value in that study was derived from a smaller set of PFTs without flattening as judged by a pulmonologist. Therefore, the “non-flattening” call was subjective. In this study, we sought to establish an objective threshold value that indicates significant flattening (flattening index, FI) in a larger set of PFTs without clinicians’ input. We determined demographic factors that may affect FI.

## Methods

### Digitization and extraction of FVL

We downloaded PFT reports in PDF format between January 1, 2015 and December 31, 2019 from the electronic medical records at Duke Health. The first 100 PFTs of each year with prebronchodilator FEV1/FVC ≥0.7, FEV1 ≥80%pred, and FVC ≥80%pred were included based on the reference equation of Crapo et al. (1981). We digitized the FVL graph and converted it to data points using a tool in Python, version 3.7 (pdfplumber; https://peps.python.org/pep-0537/) (Chen et al., 2022).

### Flattening index

**Figure 1:**
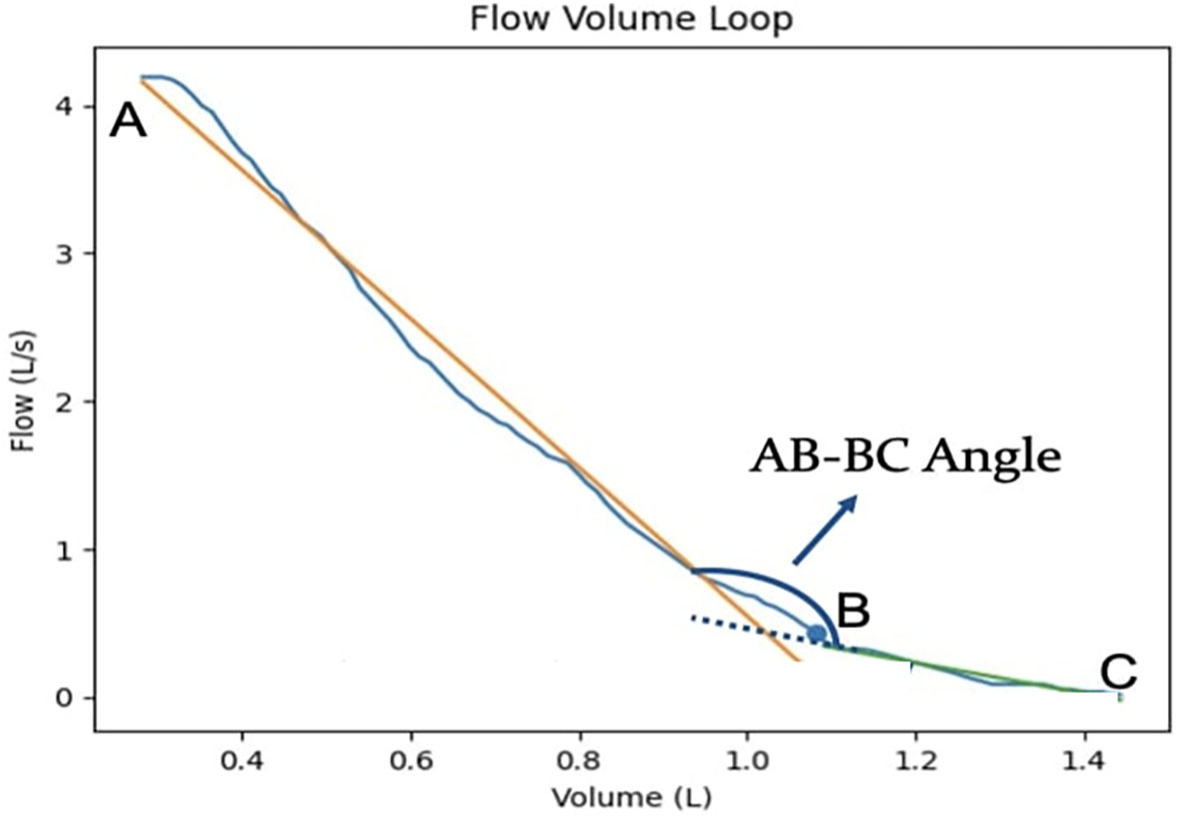
Calculation of the small airway disease index. Point A is the peak expiratory flow of the flow volume loop. Point B is the 75% point of the maximum volume. Point C is the end of the curve. Linear regression lines are generated for the segment between point A and point B (orange line) and the segment between point B and point C (dash line). The angle formed by segment AB and segment BC (AB–BC angle) is the flattening index. Adapted from Chen et al. Front Physiol. 2022 Jun 6;13:914972.

Flattening index (FI) is a measurement of the angle formed by two linear regression lines of segment AB and segment BC (**Figure 1**). Segment AB is between the peak expiratory flow (point A) and the point corresponding to 75% of the expiratory vital capacity (point B), and segment BC is between point B and the point where the expiratory portion of the FVL intersects with the x-axis (point C). Furthermore, 75% is chosen since it roughly corresponds to the start of small airways (Mead et al., 1967; Wood and Bryan, 1969; Gelb and Klein, 1977). We also extracted data on gender, age, height, and weight from PFT reports using pdfplumber 0.11.4 (https://pypi.org/project/pdfplumber/). The study was approved by the Duke Institutional Review Board (Pro00105365).

### Statistical analysis

Data were expressed as mean ± standard deviation (SD). The threshold value was defined as mean – 1.64 × SD. Multiple linear regression was performed to correlate age and BMI with FI. Student’s *t*-test was used to compare differences between two groups. *P* < 0.05 was considered statistically significant. The statistical analyses were performed using JMP Student Edition 18.2.2 (SAS Institute, Cary, NC, USA).

## Results

A total of 500 PFTs were downloaded. We excluded 10 tests that had poor quality (including poor patient effort noted by the technicians and small FVL that could not be extracted adequately by the pdf plumber to allow for accurate digitization). The final 490 PFTs were included for analysis. These tests all had at least two acceptable spirometry measurements (grades A–E according to the ATS grading system) (Graham et al., 2019).

**Figure 2:**
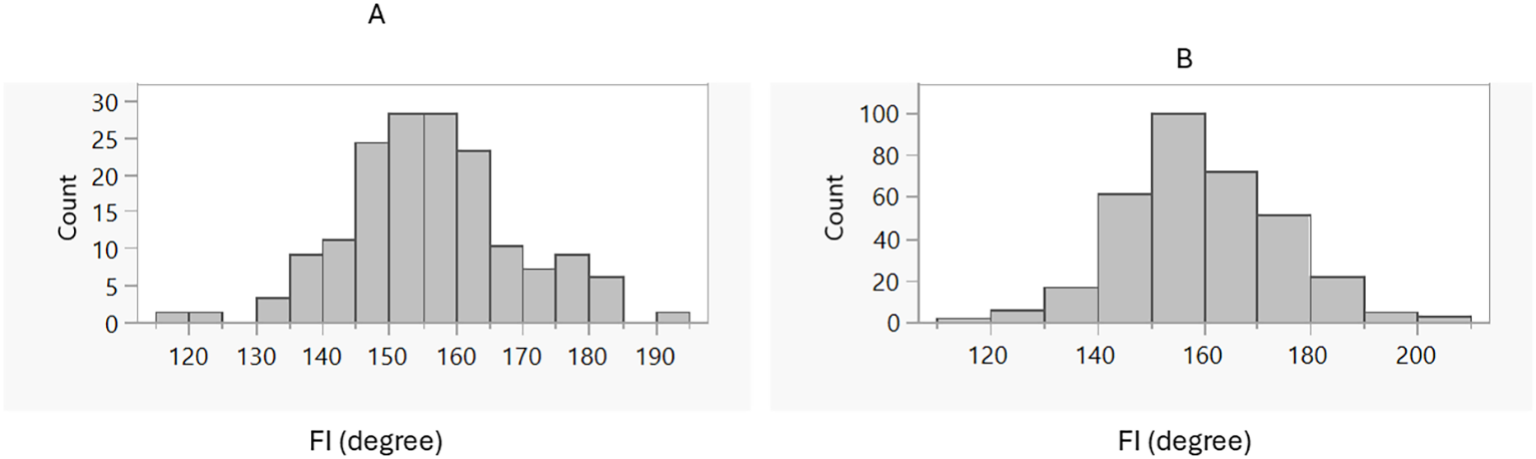
Distributions of flattening index in men **(A)** and women **(B)**. Both distributions are near Gaussian.

The FI shows a near-Gaussian distribution in men and women (**Figure 2**). The mean FI was 159° with a SD of 14°. There were 161 men and 329 women. The men had a smaller FI than the women (156° ± 12° in men vs. 160° ± 14° in women, *p* = 0.0079). Overall, FI had inverse correlations with age and BMI (**Table 1**).

**Table 1:** Multiple regression model correlating age and BMI with flattening index (FI) in men and women. .

Term	Estimate	SE	*P*-value
Men			
Intercept	196.22	7.84	<0.0001
Age	-0.35	0.09	0.0001
BMI (kg/m^2^)	-0.56	0.17	0.001
Women			
Intercept	190.90	4.46	<0.0001
Age (year)	-0.43	0.106	<0.0001
BMI (kg/m^2^)	-0.18	0.08	0.0223

R2 for men was 0.132, and R2 for women was 0.140.

SE, standard error.

The threshold value for the entire population was 136° (136°for men and 137°for women). Using this threshold, there were 21 PFTs (4.3%) in 15 patients with FI <136° (M/F = 1/2.8 vs. 1/2.0 for patients with normal FI). Their age was 63 ± 10 years (vs. 61 ± 12 years for patients with normal FI). The height was 165 ± 10 cm (vs. 167 ± 10 cm for patients with FI ≥136°), and the weight was 90 ± 21 kg (vs. 90 ± 23 kg for patients with FI ≥136°). All 15 patients with FI <136° had CT findings consistent with small airway disease and/or lung disease involving small airways (**Table 2**).

**Table 2:** Clinical characteristics of patients with abnormal flattening index (FI).

Patient	Age	Gender	FI (degree)	Pulmonary diagnosis	CT chest findings
A	70	F	135.7	ILD	Mosaic attenuation, bronchiectasis
B	67	F	132.3	Small airway disease	Scattered ground glass opacities, air trapping
C	78	F	122.2	ILD	Subpleural reticular opacities with traction bronchiectasis; scattered air trapping
D	54	M	133.8	Emphysema	Biapical paraseptal emphysema; mild ground glass opacities in both lungs
E	59	M	135.3	ILD	Diffuse ground glass opacities with air-trapping in the lower lobes
F	75	M	130.8	Lung transplant	Debris within the upper and lower bronchial tree of left lung; near-complete collapse of left lower lobe and partial atelectasis at the left upper lobe; mild upper lobe predominant centrilobular and paraseptal emphysema
G	67	F	132.5	ILD	Diffuse subpleural reticulation, traction bronchiectasis, and mosaicism
H	70	F	133.0	Asthma	Mosaic attenuation; pulmonary nodules
I	64	F	129.0	ILD	Reticular opacity and mild bronchiolectasis; mosaic attenuation
J	79	M	116.5	Hypersensitivity pneumonitis	Ground glass opacities with mosaic attenuation
K	42	F	113.5	Lung transplant	Left lower lobe consolidation with scattered linear opacities; mosaic attenuation in the right upper lobe.
L	65	F	134.9	Emphysema	Lobe predominant mild centrilobular and paraseptal emphysema; bibasilar mosaic attenuation
M	59	F	133.3	Asthma	Ground glass opacities
N	74	F	124.6	DIPNECH	Multiple pulmonary nodules; mosaic attenuation
O	48	F	122.9	Hypersensitivity pneumonitis	Ground glass opacities; air trapping

CT scans were all within 6 months of the PFTs.

ILD, interstitial lung disease; COPD, chronic obstructive pulmonary disease; DIPNECH, diffuse idiopathic pulmonary neuroendocrine cell hyperplasia.

## Discussion

In this study, we found that FI was normally distributed in men and women. It correlated negatively with age in both men and women. This is consistent with the development of small airway disease with aging (Martinez et al., 2017; Hedenstierna et al., 2019; Dai et al., 2022). FI also negatively correlated with BMI. High BMI (obesity) tends to cause small airway disease by increasing the mechanical pressure on the chest and causing low-grade systemic inflammation, which narrows peripheral airways, reduces functional residual capacity, and increases airflow resistance (Salome et al., 2008; Al-Alwan et al., 2014; Chapman et al., 2014).

The flow at the late expiratory portion of the FVL occurs in the small airways, which is less effort dependent (Mead et al., 1967; Macdonald and Cole, 1980; Menkes et al., 1981). Studies have shown that the flow in this portion of the curve is less affected by breathing a helium–oxygen mixture, supporting the contention that this portion is from the small airways where the flow tends to be laminar and thus less density-dependent (Fox et al., 1974; Macdonald and Cole, 1980; Konstantinos Katsoulis et al., 2016). Changes in other parameters have been considered to infer small airway dysfunction (Mcnulty and Usmani, 2014)—for example, increased RV/TLC indicates air trapping, but it is not specific for small airway disease (Sorkness et al., 2008). A decrease in the forced expiratory flow at 25%–75% of FVC (FEF_25%–75%_) is commonly used to infer small airway disease, but FEF_25%–75%_ is highly variable depending on the FVC and expiratory effort (Mcfadden and Linden, 1972; Boggs et al., 1982; Tager et al., 1998). The ratio of the airflow rate of 50% vital capacity to the airflow rate of 25% vital capacity (V_50_/V_25_) correlated with R_5_–R_20_ values, a measure of peripheral airway resistance in impulse oscillometry (Suzuki et al., 2015). The R_5_–R_20_ values, however, lack widespread clinical standardization and anatomic validation, making interpretation difficult across different patient populations and device types. The forced expiratory flow at 50% FVC (FEF_50%_) was reduced in asymptomatic smokers who likely had small airway disease (Dosman et al., 1975). A FEF_50%_ value less than 70% identified about 45% of patients with cough-variant asthma, a small airway disease asthma phenotype (Yuan et al., 2019). Like FEF_25%–75%_, FEF_50%_ is also dependent on expiratory effort.

The current study extended the findings from a previous study from our group that used the FVL data to develop an objective measure to quantify the degree of flattening (Chen et al., 2022). The cutoff of 149.7° in our previous study was derived from a smaller set of “non-flattening” FVL judged by a pulmonologist using mean–SD. Our current study used an objective value (mean—1.64SD) to define flattening without clinician input. If mean–SD is used in the current study, the value would be 145°, close to 149.7°. Note that the average FI in the “flattening” group in the previous study was about 145°, indicating that the clinician’s visual assessment of flattening is more liberal.

The approach to use more data in FVL to supplement the assessment of airway disease was also investigated in a previous study (Bhatt et al., 2018). The study showed new parameters (parameter D, transition point, and transition distance) derived from analyzing the FVL and volume–time curve that could identify mild airway obstruction in COPD patients who had structural abnormalities in CT scan but non-obstructive numeric FEV1/FVC values. Unlike FI in our study, these parameters were not specific for small airway disease. Note that our study also found two patients with emphysema whose numeric FEV1/FVC values did not meet airway obstruction. Therefore, both studies provided novel parameters from the graphic analysis of FVL that can identify additional patients with airway disease.

There were 21 PFTs belonging to 15 patients with FI < threshold value. These patients all had CT imaging consistent with small airway disease and/or lung diseases known to involve primarily small airways. Compared to other PFT parameters that have been used to imply small airway disease (Dosman et al., 1975; Sorkness et al., 2008; Yuan et al., 2019), FI seemed to have higher specificity in identifying patients with lung diseases characterized by small airway dysfunction using the threshold value of 136°. Our previous study used a larger threshold value (149.7°) and showed that approximately 50% of the subjects had CT abnormalities. This is not surprising since the specificity of the FI depends on the threshold value chosen. A smaller value will increase the specificity. FI would be particularly helpful for clinicians in the clinic setting in identifying patients with potential small airway disease and prioritizing further diagnostic workup, such as oscillometry and CT imaging, and treatment.

The PFTs used in the current study were done before the GLI and LLN method was proposed. Thus, the fixed ratio was used to determine significant proximal airway obstruction. In the literature, the fixed ratio underdiagnoses airway obstruction in approximately 5% of young adults compared to the LLN criteria (Cerveri et al., 2008). In older adults, 5%–10% meet the 0.70 threshold for airway obstruction but not the LLN criteria (Pothirat et al., 2015; Calverley et al., 2018). Therefore, if we had used the LLN method, we would have excluded a small number of young adults, but most of the older subjects would still have been included because the LLN usually is lower than 0.70. The correlations between age and BMI and the FI would likely still be present if the LLN method were used.

In our study, the measurement of FI requires extracting data points from the FVL graph using pdfplumber. The tools we used in this study are all free to the public. We automated the processes to fit our PFT report format, but these processes can be customized for any PFT reports in PDF format, even with a layout different from ours. Because pdf is usually a universal format for final PFT reports, our procedure focusing on the PDF format can cover reports generated from different raw data platforms that are proprietary to different vendors. However, the precision of the modeling requires that the PFT reports contain reasonably good-quality FVLs. Therefore, clinician discretion remains an essential element in the interpretation. In addition, the FI will be affected if the inflection point is not at 75% FVC. Mathematically, if the inflection point is smaller than 75% (e.g., at 65%), the FI would be bigger. To determine the variability of the inflection point will require additional mathematical modeling and can be done in future studies.

## Conclusions

FI objectively quantifies the late flattening of the FVL. It correlates inversely with age and BMI. An abnormal FI (less than the threshold value of 136°) may help identify lung diseases with small airway dysfunction. If incorporated into the PFT reports, FI can be a useful measure to assist clinicians in identifying small airway disease.

## Data Availability

The data analyzed in this study is subject to the following licenses/restrictions: It contains PHI. The de-identified version can be made available upon request. Requests to access these datasets should be directed to yuhchin.huang@duke.edu.
